# Coulomb-bound four- and five-particle intervalley states in an atomically-thin semiconductor

**DOI:** 10.1038/s41467-018-05558-x

**Published:** 2018-09-13

**Authors:** Shao-Yu Chen, Thomas Goldstein, Takashi Taniguchi, Kenji Watanabe, Jun Yan

**Affiliations:** 1 Department of Physics, University of Massachusetts, Amherst, MA 01003 USA; 2 National Institute of Materials Science, 1-1 Namiki, Tsukuba, Ibaraki 305-0044 Japan

## Abstract

As hosts for tightly-bound electron-hole pairs carrying quantized angular momentum, atomically-thin semiconductors of transition metal dichalcogenides (TMDCs) provide an appealing platform for optically addressing the valley degree of freedom. In particular, the valleytronic properties of neutral and charged excitons in these systems have been widely investigated. Meanwhile, correlated quantum states involving more particles are still elusive and controversial despite recent efforts. Here, we present experimental evidence for four-particle biexcitons and five-particle exciton-trions in high-quality monolayer tungsten diselenide. Through charge doping, thermal activation, and magnetic-field tuning measurements, we determine that the biexciton and the exciton-trion are bound with respect to the bright exciton and the trion, respectively. Further, both the biexciton and the exciton-trion are intervalley complexes involving dark excitons, giving rise to emissions with large, negative valley polarization in contrast to that of the two-particle excitons. Our studies provide opportunities for building valleytronic quantum devices harnessing high-order TMDC excitations.

## Abstract (web summary)

Valleytronic devices of atomically thin transition metal dichalcogenides may benefit from high-order exciton complexes. Here, the authors present experimental evidence for four-particle biexcitons and five-particle exciton-trions in high-quality monolayer WSe_2_.

## Introduction

Many-body correlation is a fascinating topic that has attracted decades of experimental and theoretical investigation. In atomic physics, the positronium molecule, consisting of two electrons and two positrons bound together, took more than half a century to confirm after the discovery of the positronium atom^1^. In condensed matter systems where the positrons are replaced by holes (missing electronic states in the valence band), first-principle ab initio simulations addressing complexes with three or more charged particles are challenging to implement and are being actively pursued^2–4^. Experimentally light emission due to exciton molecules, or biexcitons, is only revealed in a limited number of systems, such as carbonnanotubes, GaAs, CuCl, and GaN^5–8^.

In this work, we report the experimental observation of optical features due to biexcitons confined to a two-dimensional (2D) semiconductor, the monolayer (1L) tungsten diselenide (WSe_2_), a type of transition metal dichalcogenide (TMDC). Interestingly we discover that the biexciton consists of a spin-zero bright exciton in one valley and a spin-one ‘‘dark’’ exciton in the other. Such valley-spin configuration of the exciton molecule is unusual and has not been observed in other systems. Our samples also emit a lower-energy nonlinear feature, which we attribute to a five-particle bound state consisting of a bright trion and a dark exciton residing in two different valleys. As a result of their unique spin and valley configurations, both the biexciton and the exciton-trion produce emissions with valley polarization that has an opposite sign compared to the well-known bright exciton in a magnetic field^9,10^, and the degree of the valley polarization is much larger. As such, these multi-particle states are distinct from four-wave-mixing (FWM) measurements on MoSe_2_ and WSe_2_ in which the neutral and charged biexcitons do not involve the dark exciton^4,11^. Our findings shed light on many-body physics of transition metal dichalcogenides, and pave way for developing valleytronic devices and spin-valley entangled photon sources based on TMDCs.

## Results

### Luminescent emission due to many-body correlated states in 1L-WSe_2_

**Fig. 1:**
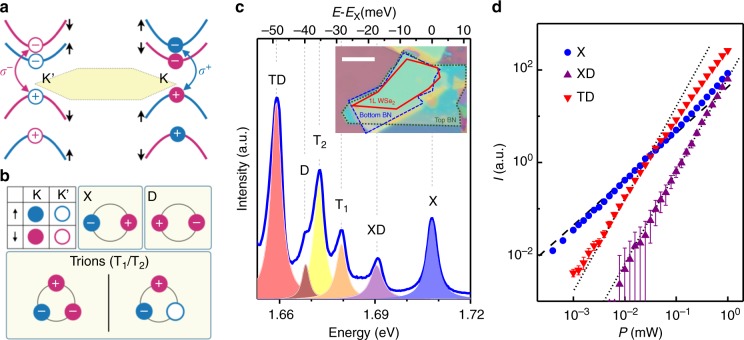
Multi-particle bound states in 1L-WSe_2._ **a** Conduction and valence band configurations at the K and K’ valleys. The color for the bands reflects the spin of the electron states. **b** The schematics of valley-spin configurations of bound states. We use blue (red) to denote spin up (down) and closed (open) symbols to represent K (K’) valley. Illustrated are the bright exciton (X), the dark exciton (D) and the negatively charged trions (T_1_/T_2_). Only states with holes in the K valley upper valence band are shown here. **c** A typical luminescence spectrum of high-quality 1L-WSe_2_ excited by 2.33 eV laser at 4 K. Inset: The OM image of the sandwiched hBN/1L-WSe_2_/hBN heterostructure. The scale bar is 10 μm. **d** The power-dependent intensity of X, XD, and TD emissions. The dashed (dot) lines in the figure are drawn for $P \propto I$ $\left( {P \propto I^2} \right)$

Monolayer hexagonal WSe_2_ is an atomically thin semiconductor with its low energy electronic states located near the K and K’ corner points—the two distinct valleys—of the Brillouin zone, suitable for developing valleytronic devices^12^. The spin of the electrons and holes in the non-centrosymmetric system is further coupled to the valley degree of freedom, and the spin degeneracy at each individual valley is lifted by spin-orbit interaction (Fig. 1a). This elegant band structure results in versatile spin-valley configurations for low-energy multi-particle bound-states in the crystal: 8 for the two-particle exciton species and 12 for the three-particle negative trion species (see Fig. 1b and Supplementary Note 1). The excitons and trions can further couple to make four- and five-particle bound states, whose underlying physics is so far not well-understood, and is the focus of this work.

The variety of spin, valley and number of particles that can form energetically well-resolved bound states results in rich light-matter interaction effects and spectroscopic features in 1L-WSe_2_, offering a variety of useful tools for accessing the quantized valley pseudospin^13^. Figure 1c shows a typical luminescence emission spectrum from our high-quality hexagonal boron nitride (hBN) sandwiched 1L-WSe_2_ sample (optical micrograph in the inset; see Methods and Supplementary Note 2 for sample preparation). We observe six intrinsic emission features from the sample in the energy range of 1.65–1.72 eV. The peak with the highest energy is the two-particle bright exciton X (full width at half maximum (FWHM): 3.4 meV, which is among the narrowest for WSe_2_ monolayers^14–16^), consisting of an electron and a hole residing in the same valley with opposite spins (top middle subpanel of Fig. 1b; note that the hole’s spin is opposite to the missing electron’s spin in the valence band). This spin-zero bound state carries opposite angular momentum of $\pm \hbar$ in the K and K’ valleys, giving rise to valley-selective coupling with circularly-polarized optical excitation^12^, a fascinating property that has been harnessed to demonstrate valley polarization as well as valley coherence in 1L-WSe_2_^17^. The bright exciton can further bind an electron in the same or the opposite valley to form trions (Fig. 1b, bottom subpanel). Our as-made sample is slightly electron doped, and we observe T_1_ and T_2_ emissions at 29 and 36 meV below X (FWHM: 3.6 and 3.7 meV for T_1_ and T_2,_ respectively), attributable to the negatively charged trions^18^.

While being the most prominent and well-known optical feature in TMDCs^19–21^, it is important to note that energetically in 1L-WSe_2_, the bright exciton X is not the two-particle ground state of the system due to the particular spin ordering of the conduction band with respect to that of the valence band (Fig. 1a)^22^. Instead, the dark exciton D illustrated in Fig. 1b upper-right subpanel, in which the electron and hole have the same spin and reside in the same valley, is energetically more favorable^23^. In the out-of-plane direction, the dark exciton is optically silent (hence the name). However, with finite in-plane magnetic field^24,25^ or momentum^26^ D becomes visible. While our experimental set-up is in back-scattering geometry (see Methods and Supplementary Note 3), the finite collection solid angle (numerical aperture NA = 0.35) and the high quality of our sample enabled us to observe this optical feature in Fig. 1c, located at about 40 meV below X (FWHM: 2.0 meV), as a result of the conduction band splitting and the distinct many-body interactions^24^.

The remainder two emission features in Fig. 1c, denoted as XD at 18 meV and TD at 49 meV below X (FWHM: 3.6 and 3.9 meV for XD and TD, respectively), are assigned as the biexciton four-particle state and the exciton-trion five-particle state, respectively. Figure 1d compares the intensity (*I*) of X, XD, and TD as a function of incident laser power (*P*) in log–log scale. In contrast to X whose intensity is nearly proportional to the incident power, both XD and TD intensities rise more steeply (black dashed and dotted lines in Fig. 1d are drawn for $P \propto I$ and $P \propto I^2$, respectively). This is similar to the case of biexcitons in other systems^5–8^, providing a first evidence that they arise from higher order complexes in the system. Meanwhile it is quite extraordinary that these nonlinear features are readily observable with continuous wave (cw) laser excitation as low as 10 µW.

### Electrostatic tuning of the many-body states

**Fig. 2:**
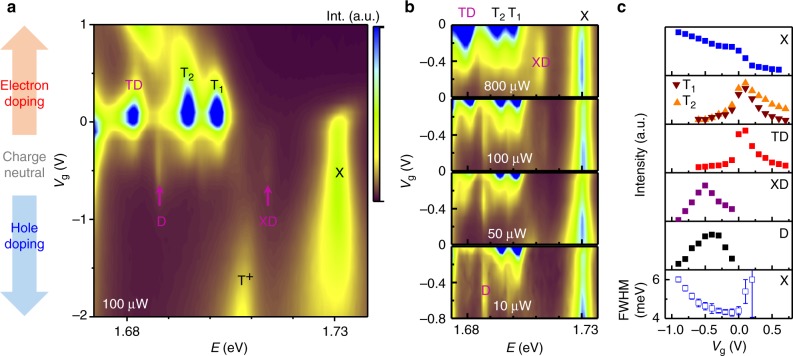
Charge doping control of the TD and XD multi-particle states. **a** Color map of PL spectra at 4 K plotted as a function of gate voltage. **b** The zoom-in color maps of PL spectra near the charge neutral region under various excitation power levels. **c** Gate-dependent intensity of corresponding peaks denoted in **a** and **b**. The bottom subpanel shows the gate-dependent FWHM of X

To gain further insights into the nature of XD and TD, we fabricate a field effect transistor (FET) device using a graphene back gate, and investigate the gate voltage dependence of its luminescence. Figure 2a displays gate voltage and emission energy mapping of the luminescence intensity at 100 µW excitation power over a gate voltage range from −2 to 1 V; data from −0.8 to 0 V at several different excitation levels of power are shown in Fig. 2b. It is evident from these mappings that all the emission features are highly sensitive to charge doping.

Similar to the sample in Fig. 1, our FET device also has minor electron doping at *V*_g_ = 0 V, and we observe strong T_1_, T_2_, and TD emission features in the absence of any gate induced charge carriers. As we remove electrons from the crystal by applying a negative gate voltage, T_1_, T_2_, and TD rapidly decrease in intensity while X becomes stronger as the sample becomes more charge neutral. Concomitantly, the dark exciton D and the biexciton XD also become prominent at low and high laser powers, respectively, for −0.6 V < *V*_g_ < −0.2 V (Fig. 2b, bottom and top subpanels). At even more negative gate voltages, D and XD disappear, and X becomes significantly broadened, accompanied by the appearance of a emission peak at about 1.71 eV attributable to the positive trion excitation (T^+^), indicating that the sample is doped by holes in this gate voltage range^17^. These observations suggest that XD is a charge neutral entity while TD is associated with electron doping.

To be more quantitative, we have extracted the intensity of various emission features as a function of gate voltage in Fig. 2c. XD is found to appear only when the X linewidth is narrow and its intensity scales with that of D. Combined with the fact that XD is a charge neutral nonlinear optical feature, we attribute it to be a biexciton consisting of a bright exciton and a dark exciton. This assignment is distinct from previous FWM measurements^4,11^ where two bright excitons are involved. Our biexciton is unlikely to arise from the binding of two X excitons. For cw excitation power of about 10 µW focused to a 2 µm spot, assuming an absorption of about 10% and X exciton lifetime of 2 ps^27,28^, the bright exciton density is estimated to be 1.7 × 10^8^ cm^−2^. Equivalently, the average X–X separation is 0.77 µm, which gives little chance for the bright excitons to meet each other before decaying through other channels. The dark exciton, on the other hand, is the lowest energy 2-particle state in the system (the intervalley version of D has the same kinetic energy, but the exchange interaction raises its energy by ~10 meV above D), and its lifetime is several orders of magnitude longer than that of the bright exciton^29^. It is thus quite reasonable to conjecture that multi-particle bound states prefer to involve D excitons at low temperatures. We rule out the possibility of the XD emission to be two D excitons bound together, since the emission energy is higher than the D exciton, which would otherwise suggest a negative binding—a state that is energetically unfavorable. The assignment of XD as a charge-neutral biexciton is further supported by theoretical calculations. Several independent simulations have consistently found that the biexciton binding energy in WSe_2_ is about 18–20meV^30–34^, which agrees well with our observed XD to be ~18 meV below X.

The physical nature of the TD emission peak is currently controversial. A previous study of 1L-WSe_2_ has observed a similar nonlinear emission feature in samples without hBN sandwiching, and has attributed it to the biexciton^35^. This assignment however, is being debated due to inconsistency with theoretical calculations, in particular the anomalously large binding energy^30–34^. From our data in Fig. 2c, the intensity of TD follows the rising and lowering of the intensities of T_1_ and T_2_, suggesting that the underlying excitation is linked to the trions. In light of the fact that D is the two-particle ground state as discussed above and that TD is observable with cw laser excitation power as low as 1 µW (Fig. 1d), we conjecture that TD results from the binding of a trion with a dark exciton. Theoretically, the binding energy of the exciton-trion five-particle state in 1L-WSe_2_ has been calculated to be ~12–15 meV^32^. We note that if we count the TD binding energy from the X emission, the value of 49 meV is three to four times too large compared to the theory. However, we believe this is not a legitimate counting since the TD complex does not involve X directly. Instead, the TD binding energy should be given by $\Delta _{{\mathrm{TD}}} = E_{\mathrm{T}} + E_{\mathrm{D}} - E_{{\mathrm{TD}}}$. We assume that the emission we observe is due to the dissociation of TD into a dark exciton and a trion, which radiatively recombines subsequently. Thus, the emission energy is given by $\hbar \omega _{{\mathrm{TD}}} = E_{{\mathrm{TD}}} - E_{\mathrm{D}} = E_{\mathrm{T}} - \Delta _{{\mathrm{TD}}} = \hbar \omega _{\mathrm{T}} - \Delta _{{\mathrm{TD}}}$, i.e., the TD binding energy needs to be counted from the trion emission energy. Indeed, the energy separation between TD and T_2_ is 13 meV, in excellent agreement with theoretical calculations.

### Thermal disassociation of the biexciton and exciton-trion bound states

**Fig. 3:**
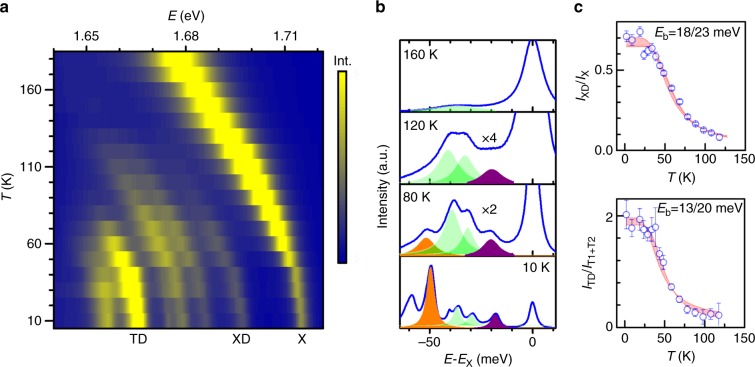
Temperature dependence of the excitonic emission. **a** Color map of PL spectra for temperatures ranging from 4 to 180 K. **b** Selected spectra at different temperatures showing the evolution of XD (purple) and TD (orange) emissions. **c** The normalized intensity of XD and TD plotted as a function of temperature. The decrease of intensity reveals the thermally activated dissociation as determined by the binding energy

The binding energies of XD and TD can be further assessed from thermal activation measurements. In Fig. 3, we plot the temperature dependence of 1L-WSe_2_ luminescence. The XD and TD peaks are found to be highly sensitive to sample heating and they disappear in the temperature range of 100 to 130 K. In contrast, the neutral exciton and the negative trion emission features survive to much higher temperatures. The comparatively more robust trion emission suggests that the binding energies of both XD and TD are smaller than that of the trions, further challenging the speculation that the TD peak is bound with respect to X.

Quantitatively the temperature dependence of XD and TD intensities are impacted by both the formation and the disassociation dynamics of these highly correlated complexes. The D exciton is the lowest energy state in the system; for temperatures below 130 K, we can assume that there are plenty of dark excitons in the crystal. This is reflected in the dramatic dropping of X intensity at low temperatures^23,36^, as well as our observation of relatively strong dark exciton emission in a backscattering optical set-up with relatively small NA. The formation process can thus be assumed to be determined by the population of the minority species, namely XD by X and TD by T in the system, which we approximate by the luminescence emission intensity of the neutral and charged excitons. By normalizing the intensity of XD and TD to the intensity of X and T, respectively, we quantitatively characterize the thermal dissociation of XD and TD as a function of temperature in Fig. 3c. This thermally activated disassociation can be captured by using the thermal activation equation considering only one binding energy:

1 $$I = \frac{{I_0}}{{1 + A{\mathrm{e}}^{ - \frac{{E_{\mathrm{b}}}}{{k_{\mathrm{B}}T}}}}},$$

where *I*_0_ is the intensity at 0 K, *E*_b_ is the binding energy, *k*_B_ is the Boltzmann constant, and *A* is a fitting parameter. Using Eq. (1) to fit our experimental data, we find that the binding energy of XD and TD to be 18–23 and 13–20 meV respectively. These values are in reasonable agreement with the theoretical calculations^30–34^ as well as the binding energy counting alluded above.

### Valleytronic properties of the biexciton and the exciton-trion

The biexciton and the exciton-trion complexes that we observed possess remarkable valleytronic properties. In the presence of time reversal symmetry, the electronic states at the K and K’ valleys are degenerate. This degeneracy is lifted in an out-of-plane magnetic field since electronic states in the two valleys have opposite magnetic dipole moment^9,10^. The resulting Zeeman splitting then leads to valley polarized charge distribution, similar to the imbalanced spin occupation in a magnetic material, which can be monitored by the optical response of the system to photons of opposite circular polarization by virtue of the valley-helicity optical selection rules^12^.

**Fig. 4:**
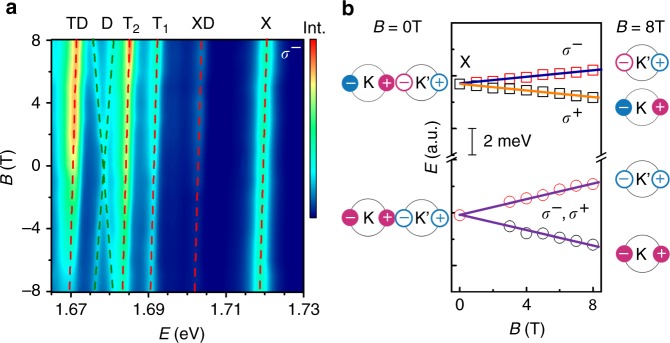
Magnetic field tuning of the XD and TD emission. **a** The color map of *σ*^−^ PL spectra in a perpendicular magnetic field from −8 to 8 Tesla. **b** Magnetic field dependence of X and D Zeeman splitting. The K’ valley states, degenerate with K states at zero fields, have higher energies at *B* = 8 T. The symbols are extracted from data in **a**. Note that the X emission is valley-helicity locked, while the D emission is unlocked

To understand the impact of valley degeneracy breaking on the four- and five-particle states, we use linearly-polarized laser light at 2.33 eV to excite our sample placed in an out-of-plane magnetic field, and collect the resultant luminescent emission in a circular-polarization resolved optical spectroscopy set-up^37^ (see Methods and Supplementary Note 3). Figure 4a shows the intensity map of *σ*^−^ luminescence for magnetic fields ranging from −8 to 8 Tesla (*σ*^+^ luminescence is displayed in Supplementary Note 4). Both XD and TD emissions obey well the valley-helicity selection rule, namely, only the K’ valley electron-hole recombination is allowed to occur in the *σ*^−^channel, similar to X, T_1_, and T_2_. In contrast, this valley-helicity locking is broken for the D exciton, and both K and K’ dark exciton recombination shows up in the *σ*^−^ luminescence, giving rise to the cross pattern in Fig. 4a not observed for the other five bound states. This observation reiterates the fact that the valley-helicity locking is for angular momentum that is perpendicular to the atomic layer^12^. In this direction, the dark exciton is a spin-one entity that cannot couple to the optical fields^26^. Instead, the observed D emission arises from radiation with momentum that is not perfectly perpendicular to the atomic layer; the projection of exciton spin and angular momentum to the light propagation direction allows for coupling of D in each valley to both *σ*^+^ and *σ*^−^ radiation.

The slope of the XD and TD emission energy vs. the magnetic field (i.e., the Zeeman shift) can be characterized by the *g*-factor: $E_{\mathrm{Z}} = g\mu _{\mathrm{B}}B$ where $\mu _{\mathrm{B}} = 0.058$ meV T^−1^ is the Bohr magneton. From Fig. 4a, XD and TD have the same slope as X, T_1_, and T_2_, and *g* = 2.17. For D however, the slope is larger, and its *g* is 4.58. This larger value of *g* is mostly due to the spin of the dark exciton. X is a spin-zero entity and its magnetic dipole moment is purely orbital; i.e., the composing electron and hole spin contributions cancel each other^9,10^. D on the other hand, is a spin-one bound state, and the spin contributes an additional 2*μ*_B_ to its dipole moment, making its Zeeman splitting almost twice that of X, as shown in Fig. 4b, consistent with a recent study^29^.

The valley-helicity locking and the Zeeman *g* factors of XD and TD emission exclude the physical picture where XD and TD emissions arise from radiative recombination of the disassociated dark exciton with finite in-plane momentum, and provide further evidence that the radiative emission of these four- and five-particle bound states are linked to either bright excitons or trions, supporting our interpretation of their formation and disassociation process as well as their binding energy interpretation discussed above.

Another important information regarding the valleytronic properties of XD and TD is encoded in the intensity of the Zeeman-split peaks. The off-resonance laser excitation with linear polarization we use populates both valleys of 1L-WSe_2_ equally with electrons and holes. However, due to the breaking of valley degeneracy, the formation probability of multi-particle bound states in the two valleys are non-equal and occupation of the lower energy states is preferred. The emission intensities from different channels thus reflect the degree of valley polarization of the corresponding underlying excitonic species.

**Fig. 5:**
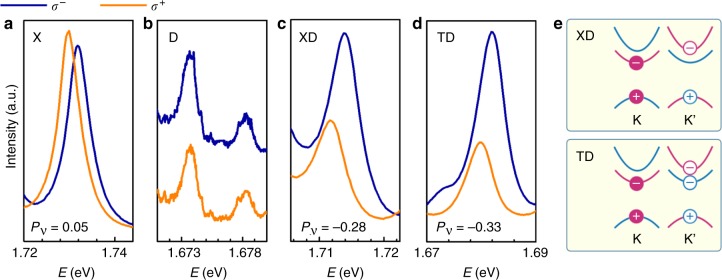
The valley polarization of excitonic bound states in a finite out-of-plane magnetic field. **a**–**d** The luminescence spectra of X, D, XD, and TD emission in *σ*^+^ and *σ*^−^ channels at 8 Tesla. Valley polarization is defined as $P_{\mathrm{V}} = \left( {I^{\sigma ^ + } - I^{\sigma ^ - }} \right){\mathrm{/}}\left( {I^{\sigma ^ + } + I^{\sigma ^ - }} \right)$. **e** The schematics of the spin-valley configuration of the XD and TD bound states

Figure 5 plots the spectral intensities of X, D, XD, and TD in *σ*^+^ and *σ*^−^ channels at 8 T. For X we observe that the *σ*^+^ emission at K valley is more intense than *σ*^−^ at K’: this is understandable since the K valley bright exciton has lower energy at positive magnetic fields. For XD and TD in Fig. 5c, d, we also observe that the *σ*^+^ emissions have lower energy than the *σ*^−^, confirming again the origin of the radiatively recombined electron and hole. What the unusual is their intensities: the lower energy *σ*^+^ emissions for XD and TD are significantly weaker than the higher energy *σ*^−^ emissions. This somewhat counter-intuitive observation is a manifestation that XD and TD are intervalley complexes, as we discuss below.

Following the convention established by previous studies^19–21^, we quantify the valley polarization by:

2 $$P_{\mathrm{V}} = \frac{{I^{\sigma ^ + } - I^{\sigma ^ - }}}{{I^{\sigma ^ + } + I^{\sigma ^ - }}},$$

where the $I^{\sigma ^ + }$ and $I^{\sigma ^ - }$ are the integrated emission intensity from the *σ*^+^ and *σ*^−^ channels, respectively. Using Eq. (2), we find *P*_V_ to be 0.05 for X, −0.28 for XD, and −0.33 for TD. The negative *P*_V_ indicates that the dark excitons involved in XD and TD reside in the opposite valley from that of X and T; see the schematic illustrations in Fig. 5e. In the presence of a magnetic field, there exist more lower Zeeman D excitons, which due to the intervalley nature of XD and TD, necessarily bind to the higher Zeeman X and T. As we discussed above, the radiation process of XD and TD involves the disassociation of the D exciton, and the X and T left behind then radiatively recombine. Hence the higher energy XD and TD emissions are more intense.

We note that using Eq. (2) for the dark exciton in Fig. 5b, one would obtain a zero *P*_V_. This reflects that in the absence of valley-helicity locking, the holy grail of TMDC valleytronics^12^, the valley degree of freedom becomes hard to access optically. By summing up the *σ*^+^ and *σ*^−^ contributions, nevertheless, the different intensities of the lower energy and higher energy Zeeman peaks *I*^L^ and *I*^H^ still reflect the population difference of the Zeeman split dark excitons, and as expected *I*^L^ is larger than *I*^H^, similar to the bright exciton X. If we define the dark exciton valley polarization as $P_{\mathrm{V}}\prime = \frac{{I^{\mathrm{L}} - I^{\mathrm{H}}}}{{I^{\mathrm{L}} + I^{\mathrm{H}}}}$, we find $P_{\mathrm{V}}\prime$ to be 0.5. This value is much larger than the 0.05 *P*_V_ for X, due to the larger Zeeman splitting of D (Fig. 4b), as well as the absence of Maialle-Silva-Sham intervalley exchange interaction^38^ that has been shown to cause valley depolarization of X^36^. It is also interesting to note that $P_{\mathrm{V}}\prime$ is larger, but reasonably close to the absolute value of *P*_V_ for XD and TD. This provides yet another evidence that D is involved in XD and TD that we observe, and its valley distribution plays a dominant role in the large valley polarization of the four- and five-particle states, as compared to the bright excitons.

## Discussion

In conclusion, we observed six intrinsic low-energy emission features arising from bound quantum states in 1L-WSe_2_. The presence of strong Coulomb interaction and the high quality of our sample enabled observation of the four-particle XD and five-particle TD bound states under a non-resonant continuous wave excitation. We assign XD as the intervalley biexciton composed of a spin-1 dark exciton and a spin-0 bright exciton, and TD as the intervalley exciton-trion consisting a spin-1 dark exciton and a negatively charged trion. These assignments may also impact the current understanding of biexciton and exciton-trion complexes in similar systems such as MoS_2_, MoSe_2_, and WS_2_ in which the assignment of biexcitons^39–41^ are also being debated. Luminescence measurements at finite magnetic fields reveal the unusual negative valley polarization for the XD and TD emission, highlighting the role of dark excitons in forming the multi-particle bound states and their intervalley nature. Our results reveal rich many-body correlated excitonic physics and pave way to applications such as those involving valley encoded quantum information.

## Methods

### Crystal growth

The bulk WSe_2_ crystals are grown by the chemical vapor transport (CVT) method. High purity W 99.99%, Se 99.999%, and I_2_ 99.99% (Sigma Aldrich) are placed in a fused silica tubing that is 300 mm long with an internal diameter of 18 mm. W and Se are kept in a 1:2 stoichiometric ratio with a total mass of 2 g. Sufficient I_2_ is added to achieve a density of 10 mg cm^−3^. The tube is pump-purged with argon gas (99.999%) for at least five times and sealed at low pressure prior to growth. Using a three-zone furnace, the reaction and growth zones are set to 1055 and 955 °C, respectively. The growth time is approximately 2 weeks.

### Sample fabrication

The atomic flakes of WSe_2_, hexagonal boron nitride (hBN) and few layer graphene (for making the FET sample) are first exfoliated on Si wafers with 300 nm of SiO_2_ and inspected under optical microscope. To make the high quality 1L-WSe_2_ heterostructures, we further use differential interference contrast (DIC) microscopy and atomic force microscopy to select residue-free flakes in order to achieve the best quality. See Supplementary Fig. 2 for typical optical micrographs taken by the DIC microscopy. The screened flakes are then stacked using a dry transfer technique with PPC (poly-propylene carbonate) stamp. All the exfoliation, inspection, and stacking processes are completed in a nitrogen purged glovebox to minimize sample degradation. The sandwiched sample is thermally annealed at 350 °C for 1 h in argon environment to improve the quality. After annealing, we can observe the significant narrowing of linewidth as well as the reduction of defect modes, as discussed in Supplementary Note 5. For the gated sample, in the stacking process we further stack a few-layer graphene piece as the back-gate electrode to tune the carrier density in the 1L-WSe_2_, using hBN as the dielectric.

### Optical/magneto-optical measurements

The sample is transferred to a closed-loop cryostat with optical access. The incident laser at 2.33 eV is reflected by a non-polarizing cube beamsplitter and then focused on the sample by a 50× objective lens (NA: 0.35) with a spot size of ~2 µm. To minimize sample heating, we keep the power <10 µW, except for power dependence measurements (see Supplementary Note 6). The luminescence signal is detected by a triple spectrometer equipped with a liquid nitrogen cooled charged coupled device (CCD) camera.

For magneto-optical measurement, we integrate the cryostat and a 9 T superconducting magnet with a room temperature bore. As illustrated in Supplementary Fig. 3. To achieve equal population at K and K’ valleys, we excite the sample with linearly polarized light at 2.33 eV. In the collection path, we first employ a quarter waveplate to transform the *σ*^−^ and *σ*^+^ helicity light into linearly polarized light with perpendicular polarization, followed by a half waveplate and a linear polarizer to resolve signals with opposite circular polarization.

### Note added to proof

We became aware of a magneto-luminescence study of WS_2_, in which a nonlinear emission peak also displays negative (or inverted) valley polarization, consistent with our results ^42^.

### Data availability

The data that support the findings of this study are available from the corresponding author on reasonable request.

## Electronic supplementary material

Supplementary Information
