# Supplementary material for: Coulomb-bound four- and five-particle intervalley states in an atomically-thin semiconductor
Source: Nat Commun. 2018 Sep 13;9:3717. doi: 10.1038/s41467-018-05558-x (PMC6137189; doi:10.1038/s41467-018-05558-x)
Supplement: Supplementary file 1 — Supplementary Information [file 41467_2018_5558_MOESM1_ESM.pdf]

Supplementary Information:

Coulomb-bound four- and five-particle intervalley states in an atomically thin semiconductor

Chen et al.

Supplementary Note 1: Spin-valley Configuration of low-energy two- and three-particle bound states in 1L-WSe_2

As illustrated in Fig.1a in the main text, the breaking of inversion symmetry as well as the strong spin-orbital coupling lifts the spin degeneracy for both the conduction band and the valence band of 1L-WSe_2. The splitting in the valence band is much larger than that in the conduction band (about one order of magnitude), about a few hundred meV[1]. In this paper, we focus on low-energy excitonic excitations that do not involve the lower valence band. To simplify the discussion, in this section we only show the three-band configuration as displayed in Supplementary Figure 1a. The notations of spin and valley follow the convention used in the main text: blue and red represent spin up and down, and filled and open symbols represent K and K' valleys respectively. Supplementary Figures 1b to 1d illustrate all possible configurations of 2- and 3-particle states in 1L-WSe_2. Restricting the hole to be located at the K valley, there are in total 4 possible configurations for the 2-particle neutral exciton states, and 6 for the 3-particle negative trion states. Swapping particles in K and K' valleys, we obtain 8 exciton and 12 negative trion configurations. For the positively charged trions, there is one hole in each valley, and there are four options for the spin and valley choice of the electron. The enlisted states are at least doubly degenerate due to the time reversal symmetry. By applying a finite perpendicular magnetic field, the valley degeneracy can be lifted as discussed in the main text.

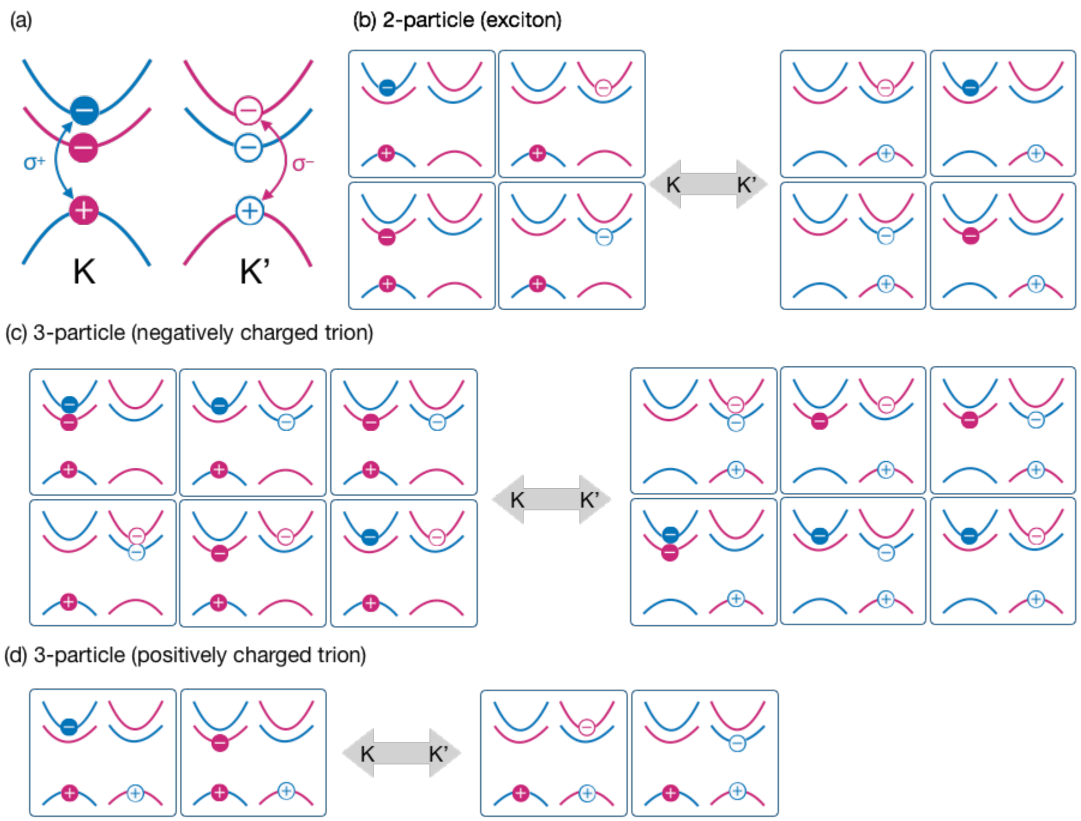

Supplementary Figure 1. (a) The three-band spin-valley configuration of 1L-WSe_2 encoded by different colors and symbols: blue (red) for spin up (down); closed (open) for K (K') valley. (b)-(d) The spin-valley configuration of 1L-WSe_2 excitons and trions. There are in total (b) 8 configurations of 2-particle exciton, (c) 12 configurations of 3-particle negatively charged trions, and (d) 4 configurations of 3-particle positively charged trions.

Supplementary Note 2: Sample Inspection by Differential Interference Contrast Microscopy

Supplementary Figure 2 shows typical optical micrographs taken by differential interference contrast (DIC) microscopy. Supplementary Figure 2a is an exfoliated few layer hBN. The tape residue can be clearly spotted. DIC microscopy can also help locating the covered flakes, enabling precise control of alignment in the transfer process. Supplementary Figure 2b demonstrates a DIC micrograph of the BN/1L-WSe_2/BN heterostructure. The sandwiched 1L-WSe_2 can be identified more easily than conventional optical microscopy.

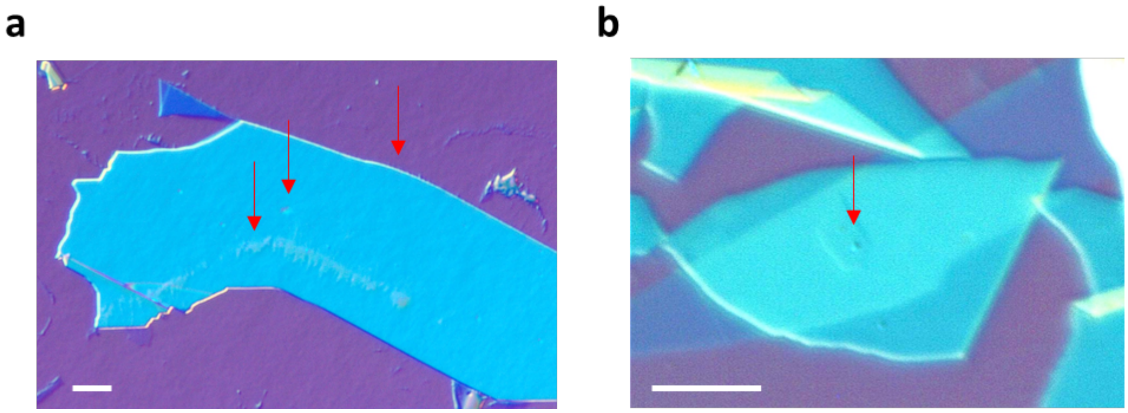

Supplementary Figure 2. (a) The DIC image of typical exfoliated few layer hBN on a SiO_2/Si substrate. The tape residues on top and edge of the hBN flake are indicated by red arrows. (b) DIC image of a BN/1L-WSe_2/BN heterostructure. The arrow points to the tiny bubble trapped on the 1L-WSe_2. The scale bar is 10 μm.

Supplementary Note 3: Optical/Magneto-Optical Measurement Setup

The optical setup used in our experiment is described in detail in the Methods section of the manuscript and is similar to the ones used in our previous work[2]. We have also included below in Supplementary Fig. 3 a schematic drawing of our magneto-optical setup. The part number is Thorlabs BS025 for the cube beamsplitter and is Olympus SLMPLN50x for the 50× objective lens. When demonstrating the nonlinearity of peak intensity as shown in Figure 1c in the main text, the heating effect is not negligible, which is reflected in the linewidth broadening and the peak redshift (see Supplementary Note 6 for detail).

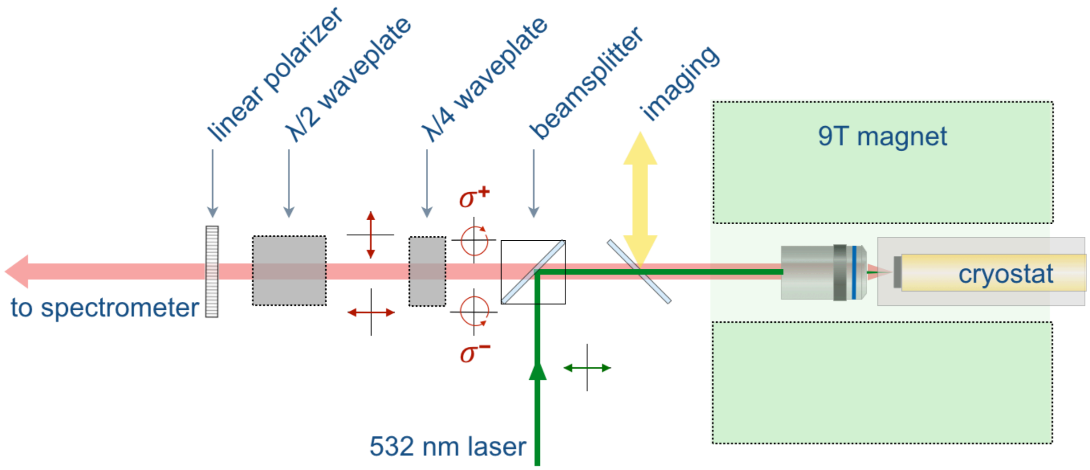

Supplementary Figure 3. The experimental setup for valley polarization measurements.

Supplementary Note 4: σ^+ Helicity Magneto-Luminescence

Complementary to PL spectra in σ^- helicity in the main text, in Supplementary Figure 4 we show the color map of magnetic field dependent PL spectra in σ^+ helicity. The opposite behaviors in energy shift and intensity profiles are consistent with the discussion in the main text, reflecting the time reversal of σ^- helicity luminescence.

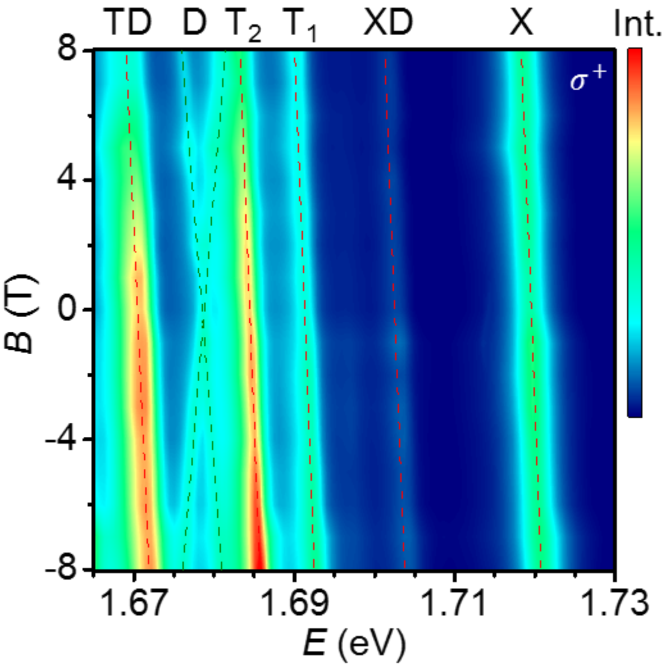

Supplementary Figure 4. The color map of σ^+ PL spectra excited at 2.33eV in a perpendicular magnetic field from -8 to 8 Tesla.

Supplementary Note 5: Defect Activated Bound States in High-Quality BN/1L-WSe_2/BN Sample

In this section, we discuss the role of defects on photoluminescence. In Supplementary Figure 5, we show the PL spectra of the BN/1L-WSe_2/BN sample under three different excitation powers. In addition to the intrinsic modes we discussed in the main text, there are several sharp features in the lower energy range. In particular, three peaks denoted as L1 to L3 that exhibit sublinear power dependence, suggesting that they originate from defect activated bound states[3]. The peak energies of L1 to L3 are in the range from 120 to 150 meV below the bright exciton (X), consistent with the Se vacancy induced bound states observed in previous studies[4].

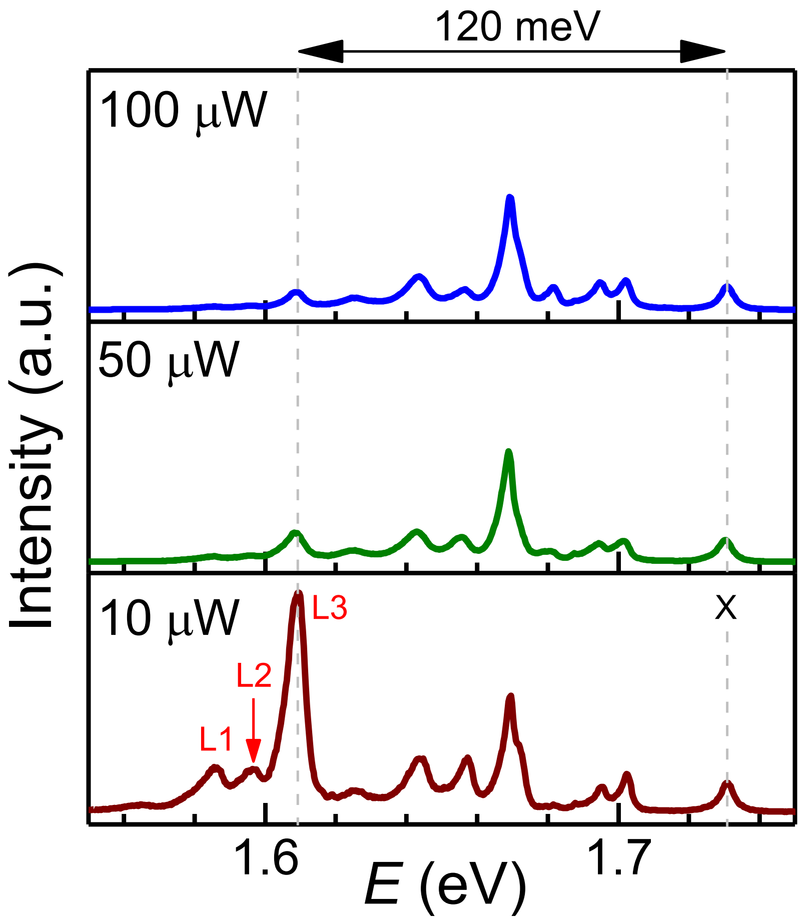

Supplementary Figure 5. The PL spectra at 4K taken at various incident laser power. The defect activated peaks, L1 to L3, are labeled. The spectra are normalized by the intensity of X.

Supplementary Note 6: Laser-Induced Sample Heating

In Figure 1d in the main text, we demonstrate the superlinear behavior of XD and TD peaks as a function of the incident power. We note that in the high-power region, there is some indication of saturation for the superlinear trends, especially for the TD emission. This effect can be understood from the cw laser power induced sample heating. To calibrate the sample temperature, we perform the temperature dependent PL from 3K to 14K with incident power at 10 μW to minimize laser-induced sample heating. The peak energy and linewidth (FWHM) are plotted in Supplementary Figure 6a and 6b as a reference for sample temperature.

Now we turn to the power dependence measurement in Figure 1d in the main text and plot the peak energy of X as a function of power. The laser-induced sample heating can be estimated by finding the peak energy shift from the X energy at 10 μW. For example, with 1 mW incident power, the peak energy of X shows a redshift about 0.25 meV, corresponding to 11.5 ± 1K in sample temperature.

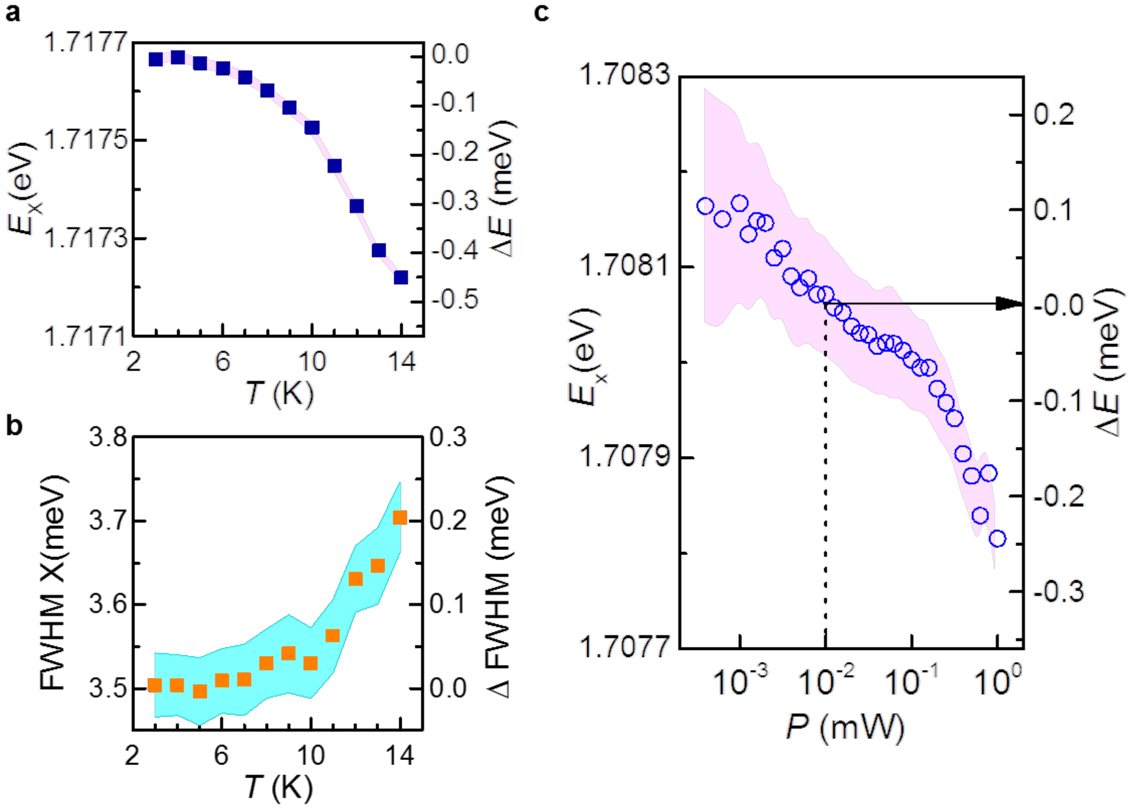

Supplementary Figure 6. (a)&(b) The temperature dependence of bright exciton peak energy (a) and FWHM (b) extracted for calibrating sample temperature. The incident power for taking the calibration spectra is 10 μW. (c) The peak energy of X plotted as a function of incident laser power.

Supplementary References:

1. Liu, G.-B. Bin, Shan, W.-Y. Y., Yao, Y., Yao, W. & Xiao, D. Three-band tight-binding model for monolayers of group-VIB transition metal dichalcogenides. *Phys. Rev. B - Condens. Matter Mater. Phys.* **88,** 1–11 (2013).
2. Chen, S.-Y., Zheng, C., Fuhrer, M. S. & Yan, J. Helicity resolved Raman scattering of MoS2, MoSe2, WS2 and WSe2 atomic layers. *Nano Lett.* **15,** 2526–2532 (2015).
3. Schmidt, T., Lischka, K. & Zulehner, W. Excitation-power dependence of the near-band-edge photoluminescence of semiconductors. *Phys. Rev. B* **45,** 8989–8994 (1992).
4. Wu, Z. *et al.* Defects as a factor limiting carrier mobility in WSe2: A spectroscopic investigation. *Nano Res.* **9,** 3622–3631 (2016).
